# An international, multicentre evaluation and description of *Burkholderia pseudomallei* infection in cystic fibrosis

**DOI:** 10.1186/s12890-015-0109-9

**Published:** 2015-10-09

**Authors:** James B. Geake, David W. Reid, Bart J. Currie, Scott C. Bell, MelioidCF Investigators

**Affiliations:** Department of Respiratory Medicine, The Lyell-McEwin Hospital, Haydown Road, Elizabeth Vale, 5112 South Australia Australia; The Prince Charles Hospital, Rode Road, Chermside, Brisbane, 4032 Australia; QIMR Berghofer Medical Research Institute, 300 Herston Road, Herston, Brisbane, 4006 Australia; Menzies School of Health Research and Royal Darwin Hospital, Darwin, NT Australia

**Keywords:** Burkholderia pseudomallei, Cystic fibrosis, Melioidosis, Patient outcome assessment, Therapeutics

## Abstract

**Background:**

Several cases of *Burkholderia pseudomallei* infection in CF have been previously reported. We aimed to identify all cases globally, risk factors for acquisition, clinical consequences, and optimal treatment strategies.

**Methods:**

We performed a literature search to identify all published cases of *B. pseudomallei* infection in CF. In addition we hand-searched respiratory journals, and contacted experts in infectious diseases and CF around the world. Supervising clinicians for identified cases were contacted and contemporaneous clinical data was requested.

**Results:**

25 culture-confirmed cases were identified. The median age at acquisition was 21 years, mean FEV_1_ % predicted was 60 %, and mean BMI was 19.5 kg/m^2^. The location of acquisition was northern Australia or south-east Asia for most. 19 patients (76 %) developed chronic infection, which was usually associated with clinical decline. Successful eradication strategies included a minimum of two weeks of intravenous ceftazidime, followed by a consolidation phase with trimethoprim/sulfamethoxazole, and this resulted in a higher chance of success when instituted early. Three cases of lung transplantation have been recorded in the setting of chronic *B. pseudomallei* infection.

**Conclusion:**

Chronic carriage of *B. pseudomallei* in patients with CF appears common after infection, in contrast to the non-CF population. This is often associated with an accelerated clinical decline. Lung transplantation has been performed in select cases of chronic *B. pseudomallei* infection.

**Electronic supplementary material:**

The online version of this article (doi:10.1186/s12890-015-0109-9) contains supplementary material, which is available to authorized users.

## Background

*Burkholderia pseudomallei* is a gram-negative environmental bacterium found in soil and surface water that causes melioidosis, which most commonly occurs in south-east Asia and northern Australia [1]. Melioidosis has been increasingly recognised to occur in diverse tropical locations globally, including the Americas and Africa [2]. *B. pseudomallei* is now classified by the US CDC as a tier-1 select agent because of its aerosolized biothreat potential (http://www.selectagents.gov/). Infection can occur through inhalation, aspiration and ingestion, although transmission most commonly occurs via percutaneous inoculation [3]. Pneumonia is the most common clinical manifestation (presumably via haematogenous spread to the lungs), with a spectrum including mild self-limiting infection, subacute pulmonary disease mimicking tuberculosis, rapidly progressive multifocal pneumonia and systemic sepsis which confers a high mortality of over 50 % [4]. Melioidosis is often reported in people returning to their home country after travelling to an endemic region. At-risk tourists include those with diabetes and cystic fibrosis (CF) exposed to soil and surface water or monsoonal storms where aerosolization of *B. pseudomallei* may occur [1, 2].

Generally patients who survive infection clear the organism and rarely relapse following an adequate duration of therapy [5]. In the 25-year Darwin Prospective Melioidosis Study [6], only one of over 750 consecutive melioidosis patients had evidence of long-term persisting infection with *B. pseudomallei*. This patient with non-CF bronchiectasis has continued to have positive sputum culture results for *B. pseudomallei* 14 years after diagnosis and treatment of melioidosis. Whole genome sequencing of isolates obtained from this patient over a 12-year period demonstrates loss of virulence and immunogenic factors, as well as deletion of pathways involved in environmental survival [7]. There have been a number of published case reports of patients with CF who have acquired *B. pseudomallei* infection and a number of these cases have demonstrated chronic carriage of *B. pseudomallei* in patients with CF [8–17].

In this study, we aimed to describe the international experience of infection with *B. pseudomallei* in patients with CF. We have examined potential risk factors for acquisition and persistence of infection, summarised global experience of effective antibiotic strategies, and assessed the long-term clinical sequelae of infection.

## Methods

The study was conducted in accordance with the amended Declaration of Helsinki, and was approved by the local human research ethics committee. (Metro North Hospital and Health Service Human Research Ethics Committee, Queensland, HREC/13/QPCH/51). A literature search (utilising Medline, Embase, CINAHL, AustHealth and ScienceDirect databases and by Google Scholar) was performed to identify all published cases of *B. pseudomallei* infection in patients with CF in July 2013 and repeated in August 2014 (Additional file 1). Case reports were hand-searched and clinicians from European, North American, and Australasian CF centres that had either reported *B. pseudomallei* in CF patients, or were experts from CF microbiology laboratories, were contacted to locate additional cases not previously reported. Data entry sheets were provided to the clinicians that had supervised the care of the identified cases, and results including contemporaneous clinical metrics were compiled (Additional file 2). Where supervising clinicians could not be contacted, information was extracted from the published reports [8, 9, 11, 14]. Chronic infection was defined as persistent cultures of *B. pseudomallei* from sputa or endobronchial washings for 12 months or more.

## Results

### Case identification

Twenty-five culture-proven cases between 1987 and 2015 were identified (Table 1). 16 cases were identified by literature search. A further nine cases were identified through international consultation with experts of CF and infectious disease that had experience in the diagnosis and management of *B. pseudomallei* infection. Of the 25 cases, detailed clinical data were made available by the treating clinicians for 21 cases, and in the remaining four cases details were extracted from published reports. An additional two cases were identified where melioidosis was suspected based on positive *B. pseudomallei* serology, although culture confirmation was elusive. Both cases died from progressive refractory respiratory infection after being presumptively infected while travelling overseas; these cases were not included further in this analysis. Three additional possible cases from Mexico were identified through hand-searching references of published articles [18]. There were insufficient data available and the accuracy of bacterial identification was uncertain, therefore these cases were also excluded.

**Table 1:** Cases of culture confirmed B. pseudomallei infection in CF

Patient	Year isolated	Location of acquisition	Country of residence	Age	Gender	CFTR Mutation	FEV_1_%	BMI	Co-pathogens	ETOH >40 g daily	Diabetes
1 (O’Carroll, 2003) [12]	1987	Northern Australia	Australia	21	M	F508del/F508del	49	21	Pseud	No	No
2	Early 1990’s	Northern Australia	Australia	40	M	F508del/R117	44.5	23	Pseud	Yes	No
									*Mycobacterium intracellulare*		
3 (Schülin, 2001) [14]	1992	Likely Thailand	Germany	31	M	Unknown	Unknown	Unknown	Unknown	Unknown	Unknown
4 (Visca, 2001) [15]	1998	South-East Asia	Italy	25	F	Unknown	Unknown	Unknown	Pseud	Unknown	Unknown
									BCp		
5 (Dance, 1999) [11]	1998	Malaysia	England	20	M	Unknown	Unknown	Unknown	Unknown	Unknown	Unknown
6 (Holland, 2002) [16]	1999	Northern Australia	New Zealand	8.5	M	G551D/1717-16 > A	46	15	Pseud	No	No
									*Aspergillus fumigatus*		
7 (Holland, 2002) [16]	2000	Northern Australia	New Zealand	7	F	G551D/1717-16 > A	Unknown	19	*Haemophilus influenzae*	No	No
									Staph A		
8 (O’Carroll, 2003) [12]	2000	Northern Australia	Australia	21	M	F508del/F508del	35	22	Pseud	No	Yes
									Staph A		
									BCp		
9^a^ (Holland, 2002) [16]	2000	Northern Australia	Australia	18	M	F508del/F508del	45	21	Pseud	Yes	No
									Staph A		
									BGl		
									*Mycobacterium intracellulare*		
									Other		
10 (O’Carroll, 2003) [12]	2001	Northern Australia	Australia	14	M	F508del/G542X	80	17	Pseud	No	No
									Staph		
11 (Holland, 2002) [16]	2001	Northern Australia	New Zealand	38	M	F508del/F508del	36	19	Pseud	No	IFG
									Staph A		
12 (O’Carroll, 2003) [12]	2001	Northern Australia	Australia	25	F	Unknown	80	20	Pseud	No	No
									Staph A		
									BCp		
13 (Asiah, 2006) [8]	2004	Malaysia	Malaysia	17	M	Unknown	Unknown	Unknown	Pseud	Unknown	Unknown
									Staph A		
14 (Barth, 2007) [9]	2005	Brazil	Brazil	17	F	Unknown	Unknown	Unknown	Pseud	Unknown	Unknown
									Staph A		
15	2005	Thailand	England	30	F	Unknown	56	21	Pseud	No	Yes
									Staph A		
									*Aspergillus fumigatus*		
16 (Corral, 2008) [10]	2006	British Virgin Islands	England	17	M	F508del/E60X	63	22	Pseud	Unknown	No
									Staph A		
			Canada								
17	2007	Northern Australia	Australia	38	M	F508del/F711 + 5G > A	29	19	Pseud	No	No
									Staph A		
									*Aspergillus terreus*		
18	2007	Thailand	New Zealand	10	F	F508del/17aa + IG74	107	16	Pseud	No	No
									Staph A		
									Other		
19	2008	Thailand	Australia	22	F	F508del/F508del	85	23	Pseud	Yes	Yes
20 (O’Sullivan, 2011) [13]	2009	Aruba	USA	7	F	F508del/F508del	88	13	None	No	No
21	2010	Vietnam/Cambodia	England	23	F	F508del/F508del	44	19	Pseud	No	No
									*Haemophilus influenzae*		
22 (Radhakrishna, 2014) [17]	2011	Thailand	Australia	25	M	Unknown	95	24	Pseud	Unknown	No
									*Aspergillus fumigatus*		
23	2012	Malaysia/Philippines	Malaysia	19	M	R553X/unknown	18	17	Pseud	No	No
24	2013	Thailand/Japan	England	30	M	F508del/E60X	67	23	Pseud	No	No
25	2015	Thailand	England	21	F	F508del/F508del	71	18	Pseud	No	No
									Staph A		

*Pseud Pseudomona aeruginosa, Staph A Staphylococcus aureus, BCp Burkholderia cepacia* complex, *BGl Burkholderia gladioli, IFG* impaired fasting glucos

^a^3 distinct *B. pseudomallei* infections were recorded in this patient over 8 years

### Epidemiology and risk factors for *B. Pseudomallei* acquisition

Of the 25 cases, 15 (62.5 %) were males, and ten females. The median age at acquisition was 21 years (range 7–38 years). The presumptive location of acquisition for the majority was either northern Australia or South-east Asia. There was one case each of presumed acquisition in Brazil, Aruba and the British Virgin Islands, respectively. The mean forced expiratory volume in 1 s (FEV_1_) was 60 % predicted (range 38-107 %) and mean body mass index (BMI) was 19.5 kg/m^2^ (range 13–24). Two cases were siblings. Specific Cystic Fibrosis Transmembrane Conductance Regulator (CFTR) mutations were documented in 17 of the 25 patients. Of these, 47 % (8) were F508del homozygotes and 35 % (6) were F508del heterozygotes. Having CF (pulmonary disease) was the apparent major risk factor in all patients. Of the 19 patients where data were available, CF-related diabetes was present in three (16 %) and there was impaired glucose tolerance in one. Other common risk factors for infection with *B. pseudomallei* such as hazardous alcohol use, chronic renal disease, congestive cardiac failure, rheumatic heart disease and immunosuppressive therapy were not reported in any of the cases.

### Clinical and radiological manifestations of infection with *B. Pseudomallei*

Of the 25 patients, clinical manifestations at the presumed time of acquisition were available in 24. In ten (42 %), isolation of *B. pseudomallei* was an incidental finding on routine sputum surveillance and in the remaining 14 patients (61 %), isolation of *B. pseudomallei* was associated with symptoms of increased cough and sputum. Systemic features (either one or a combination of the following: fever, weight loss or deteriorating glycemic control) were reported in 10 patients. New radiological changes were documented in eight patients, with patchy areas of consolidation being the most common abnormality reported. Lobar consolidation, progressive lobar destruction, increased bronchiolectasis with “tree-in-bud” opacities and progressive bronchiectasis were also reported.

### Long-term outcomes following infection with *B. Pseudomallei*

In five patients, acquisition of *B. pseudomallei* did not result in chronic infection (≥12 months). In one of these patients (patient 9), there were three different *B. pseudomallei* infections over an eight-year period, each with distinct organisms as confirmed by multilocus sequence typing, [6] and each episode was successfully cleared with 2 to 3 week courses of intravenous ceftazidime and oral trimethoprim/sulfamethoxazole (TMP/SMX) followed by consolidation with three months of oral TMP/SMX. This case was considered to have been infected with distinct *B. pseudomallei* strains on three occasions [6]. Two patients presented with acute pneumonia and the organism was eradicated after a combination of intravenous followed by consolidation nebulized and oral antibiotics (patient 16 and 20). One further patient who was not unwell at the time of isolation of the organism spontaneously cleared the infection without specific antibiotics directed at *B. pseudomallei* (patient 19).

In 19 patients (79 %)*,* initial infection was followed by evidence of chronic infection (range 1 – 11 years) (Table 2). Of these patients, 14 were thought to have had an accelerated decline in pulmonary status by their physician, although in one case the reported decline occurred after a four-year period of clinical stability despite evidence of persistent infection. Markers of clinical deterioration that were reported included increased exacerbation frequency, reduced response to intravenous antibiotics and an accelerated decline in lung function. Six patients had died by August 2014, between two and six years after the first isolation of *B. pseudomallei* (patients 6, 7, 8, 11, 15 and 18). In two, septicemia was documented and complicated by respiratory failure and death (patients 7 and 15). In one recurrent bacteraemia occurred and oral corticosteroids appeared to precipitate an episode (patient 15). Tigecycline controlled bacteremia despite progressive resistance to TMP/SMX and ceftazidime.

**Table 2:** Cases of chronic infection

Patient	Duration of infection	Year of acquisition	Eradication attempted	Eradication successful	Outcome
1 (O’Carroll, 2003) [12]	>15 years	1987	No	N/A	Slow decline in lung health consistent with CF; transplant 2004
10 (O’Carroll, 2003) [12]	11 years	2001	No	N/A	Slow decline in lung health consistent with CF
3 (Schülin, 2001) [14]	>9 years	1992	Yes	No	Increased frequency of exacerbations
15	>8 years	2005	Yes	No	Accelerated decline; transplant declined due to *B. pseudomallei* infection; *B. pseudomallei* septicaemia;^a^ died 2009
22 (Radhakrishna, 2014) [17]	7 years	2011	Yes	Yes	No obvious clinical impact
2	7 years	Early 1990’s	No	N/A	Spontaneously clearance of infection after approx. 7 years; 11 years later died of neutropenic sepsis complicating treatment of Duke’s C colon cancer.
7 (Holland, 2002) [16]	>6 years	2000	Yes	No	Accelerated decline with increased frequency of exacerbations; *B. pseudomallei* septicaemia; died 2009
17	6 years	2007	No	N/A	Slow decline in lung health consistent with CF
18	6 years	2007	Yes	No	Accelerated decline; transplant declined due to *B. pseudomallei*; died 2013
11 (Holland, 2002) [15]	5 years	2001	Yes	No	Accelerated decline; transplant 2006; died 2011
23	>12 months	2012	Yes	No	Progressive destruction right upper lobe
6 (Holland, 2002) [16]	>4 years	1999	Yes	No	After presumed latency of 4 years developed accelerated decline with pneumonia; died 2004
24	4 years	2009	Yes	Unknown	Fall in lung function; stable after targeted antimicrobial therapy
14 (Barth, 2007) [9]	>2 years	2005	Yes	No	Accelerated decline with rapid decrease in lung function and recurrent exacerbations over 2 years; long term outcome unknown
8 (O’Carroll, 2003) [12]	2 years	2000	Yes	No	Rapid decline post infection; died 2 years after initial infection
21	2 years	2010	Yes	No	Accelerated decline; transplant 2012 with persistent infection post-transplant
12 (O’Carroll, 2003) [12]	2 years	2001	Yes	Yes	Repeated admissions with pneumonia, treated with 3 week courses of ceftazidime, meropenem, tobramycin; infection ultimately spontaneously cleared
4 (Visca, 2001) [15]	1 year	1998	Yes	Yes	Deteriorating pulmonary sepsis, increasingly refractory to anti-pseudomonal antibiotics; cleared infection with antimicrobial therapy; still alive 2013
13 (Asiah, 2006) [8]	1 year	2004	Yes	Yes	Increased pulmonary sepsis during infection with *B. pseudomallei*; remained well 5 months after completing targeted anti-microbial therapy

^a^Recurrent episodes of *B. pseudomallei* bacteremia

^b^In patient 5 the duration of infection and long term outcome was unknown

In five patients, evidence of chronic infection (duration 5–17 years) was not associated with clinical deterioration (patients 1, 2, 10, 17 and 22).

In one patient duration of infection and outcome was unknown (patient 5), and another one patient was identified shortly prior to publication (patient 25). Initial sputa have been clear after three weeks of intravenous antibiotics. The patient continues on suppressive oral antibiotics, and long term outcomes are awaited.

### Eradication strategies

Specific eradication strategies were documented in 19 of the 25 patients. Six patients (including patient 9 - multiple episodes of infection) achieved clearance of *B. pseudomallei*. Eradication attempts were unsuccessful in 11 patients and there was an unknown outcome in two patients (patient 24, patient 25, Table 3). For cases of successful eradication, the period of known infection was usually less than 12 months, whereas failed eradication usually occurred where long-term infection was evident. Ceftazidime was a key component of induction therapy in all but one of the patients with successful eradication, and all had consolidation therapy from one to four months after completion of intravenous antibiotics and in most cases, oral TMP/SMX was used. In select cases, antibiotic regimens also included combinations of oral amoxicillin/clavulanic acid, doxycycline and chloramphenicol, and nebulized meropenem (250–500 mg twice daily in 4 ml of sterile water).

**Table 3:** Eradication strategies

Patient	Duration of infection prior to treatment	Induction		Consolidation	
		Treatment	Duration	Treatment	Duration
Successful eradication					
16 (Corral, 2008) [10]	Unknown – lived in BVI	Meropenem 2 g tds	19 days	TMP/SMX 960 mg bd	19 days
				Minocycline 100 mg bd	
		Minocycline 100 mg bd			
				Tobramycin (neb)^a^ 80 mg bd	
20 (O’Sullivan, 2011) [13]	3 months	Imipenem	14 days	Meropenem (neb)	28 days
		Ceftazidime		TMP/SMX 960 mg bd	2 years
4 (Visca, 2001) [15]	12 months	Ceftazidime	42 days	TMP/SMX 960 mg bd	210 days
		TMP/SMX 960 mg bd		Doxycycline	
				Chloramphenicol	
13 (Asiah, 2006) [8]	12 months	Ceftazidime	56 days	TMP/SMX 960 mg bd	112 days
		TMP/SMX 960 mg bd		Doxycycline	
22 (Radhakrishna, 2014) [17]	7 years	Meropenem 2 g tds	Approx 56 days	Doxycycline followed by amoxycillin/clavulanic acid (doxycycline allergy)	12 months
		Ceftazidime 3 g tds			
		Tobramycin (neb) 80 mg bd			
		TMP/SMX 960 mg bd			
9 (Holland, 2002) [16] Infection 1	Unknown	Ceftazidime	14 days	TMP/SMX 960 mg bd	90 days
		TMP/SMX 960 mg bd			
9 (Holland, 2002) [16] Infection 2	1 month	Ceftazidime	21 days	TMP/SMX 960 mg bd	90 days
		TMP/SMX 960 mg bd			
9 (Holland, 2002) [16] Infection 3	1-2 months	Ceftazidime	14 days	TMP/SMX 960 mg bd	90 days
		TMP/SMX 960 mg bd			
Failed Eradication					
8 (O’Carroll, 2003) [12]	4 months	Meropenem 2 g tds	56 days	None	
		Ceftazidime 3 g tds			
17	6-12 months	Ceftazidime 3 g tds	14 days	TMP/SMX 960 mg bd	84 days
		TMP/SMX 960 mg bd			
23	>10 months	Ceftazidime 3 g tds	21 days	Unknown (patient moved to Malaysia)	
		TMP/SMX 960 mg bd			
		Tobramycin 5 mg/kg			
3 (Schülin, 2001) [14]	6 years	Unspecified antipseudomonal antibiotics	14 days	No (exacerbations directed against PsA)	
14 (Barth, 2007) [9]	>18 months	Pipracillin/clavulanate	21 days	Meropenem 2 g tds	21 days
		Tobramycin 5 mg/kg		Ceftazidime 3 g tds	
				Amikacin	
				TMP/SMX 960 mg bd	
15	>8 years	Meropenem 2 g tds	14 days	TMP/SMX 960 mg bd	126 days
		Tobramycin		Minocycline 100 mg bd	
				Meropenem (neb)	
18 (1^st^ attempt)	2 months	Ceftazidime 1.5 g tds	14 days	Infection not cleared therefore 2^nd^ course of IV antibiotics	
		Tobramycin 300 mg			
		Amoxycillin/Clavulanic acid 900 mg			
18 (2^nd^ attempt)	2 months	Meropenem	84 days	TMP/SMX 960 mg bd	Long term
		Ceftazidime		Doxycycline	
		Amoxycillin/Clavulanic acid			
6 (Holland, 2002) [16]	>4 years	Ceftazidime 50 mg/kg	120 days	None (exacerbations directed against *B. pseudomallei*)	
		Piperacillin 50 mg/kg			
		Amoxycillin/Clavulanic acid			
7 (Holland, 2002) [16]	>5 years			TMP/SMX 960 mg bd	4 years
1^st^ isolation					
7 (Holland, 2002) [16]	>9 years	Imipenem	20 days	TMP/SMX 960 mg bd	19 days
2^nd^ isolation		Piperacillin/Clavulanic acid	42 days	Tetracycline	30 days
				Meropenem/TMP/SMX	>30 days
11 (Holland, 2002) [16]	3 months	Ceftazidime	14 days	TMP/SMX 960 mg bd	330 days
		Tobramycin			
21	1 month	Meropenem 2 g tds	42 days	Tobramycin (neb) 300 mg bd	42 days
		Ceftazidime 3 g tds		TMP/SMX 960 mg bd	
		Tobramycin 7 mg/kg			

Success of eradication for patient 24 and patient 25 unknown

^a^neb = nebulized

Two patients spontaneously cleared infection without specific eradication strategies (patients 2 and 19) (Table 2). Whilst not a formal approach at eradication of *B. pseudomallei*, patient 12 was treated with 3-week courses of ceftazidime, meropenem and tobramycin over a 15-month period for repeated episodes of pneumonia, after which time *B. pseudomallei* was no longer cultured from sputum.

### Transplantation with *B. pseudomallei*

Transplantation was considered for six patients but two patients were not listed due to concerns about the potential risk of *B. pseudomallei* graft infection after transplantation (patient 15, patient 18). One patient was listed for transplant but deteriorated very rapidly and died several months later without transplant (patient 8). Three patients have undergone lung transplants (patients 1, 11 and 21) and immediate post-transplant results have been satisfactory. One patient (patient 1) remains well 10 years post-transplant with normal lung function despite endobronchial washings remaining positive for *B. pseudomallei*; another patient (patient 12) died five years after transplantation, and post-transplant endobronchial washings were positive; and the third patient (patient 21) is alive two years after transplantation also with *B. pseudomallei* recurrently isolated from post-transplant respiratory samples. Chronic *B. pseudomallei* suppression has been used in all cases (either TMP/SMX or doxycycline).

## Discussion

CF is a disease that predominates in people of Caucasian descent and most patients do not live in tropical or sub-tropical regions where *B. pseudomallei* is endemic. As survival has increased, CF patients have increasingly had the opportunity to travel, and as a consequence over the past two decades there have been a number of reports of infection with *B. pseudomallei* in CF patients [8–17].

We have identified 25 cases of *B. pseudomallei* in CF patients and the vast majority of CF patients who have become infected with *B. pseudomallei* have acquired the organism through travel to endemic areas such as Southeast Asia and northern Australia. Most patients already had significant structural lung disease and low BMI, which suggests these factors are likely to be important risk factors for acquisition of the organism in CF. However severe CF disease *per se* was not an obvious prerequisite for acquisition. Some patients were also diabetic but other risk factors for melioidosis such as hazardous alcohol use, chronic renal disease, heart disease and immunosuppressive therapy were not present in this cohort.

In contrast with the general population, acquisition of *B. pseudomallei* in CF appeared to be more likely to result in chronic infection, which is problematic given how difficult it can be to eradicate this pathogen even with targeted antimicrobial therapy. Whilst the clinical manifestations of infection were varied in the CF patients, and a chronic quiescent disease state can obviously occur, the establishment of chronic infection in most patients usually heralded further clinical deterioration, with progressively refractory bronchopulmonary sepsis being a common feature. Delayed diagnosis may contribute to chronic infection and early attempts to eradicate are recommended. This requires expeditious recognition of the infection in travellers returning from endemic areas. The identification of *B. pseudomallei* can be challenging, and it is possible that isolates may be confused with other more common CF pathogens including other Burkholderia species, particularly if laboratories are not familiar with *in vitro* characteristics of *B. pseudomallei* [8, 14, 15]. Furthermore, infection with *B. pseudomallei* does not always result in immediate onset of symptoms as it often would in patients without CF, and symptoms may masquerade as those typical of pulmonary exacerbation. It is therefore important that CF clinicians have a high index of suspicion for patients returning from endemic areas.

Whilst not universally successful, approaches that utilise a 2–3 week course of parenteral ceftazidime, followed by a 3-month consolidation course of oral.

TMP/SMX, appear to be the most effective. This approach is similar to that recommended for melioidosis therapy [1, 2, 19]. If initial response does not successfully result in persistently negative sputum cultures for *B. pseudomallei,* then a longer course of parenteral ceftazidime or meropenem (≥4 weeks) should be considered [2]. If patients are allergic to or intolerant of TMP/SMX, consolidation with several months of doxycycline or amoxycillin/clavulanic acid have also been successfully used. When eradication is not possible, and a progressive decline in health ensues, lung transplantation may be considered. To date, early post-transplant outcomes have been acceptable despite persistence of *B. pseudomallei*.

This case series has several limitations. Firstly, it is likely other cases have not been identified, either because they have not been reported, or the infection was not recognised. For example, we excluded three possible cases from Mexico because of insufficient details [18]. Secondly, outcomes were not available for some patients, we were unable to access data (patients 5, 13 and 14), or cases lost to follow-up (patients 16 and 23). Interestingly, two patients were reported by two CF centres, by two UK-based centres, and by centres in the UK and Canada, respectively (patients 15 and 16). After further investigation we noted they were the same persons, highlighting the mobility of CF patients.

## Conclusion

The international experience with *B. pseudomallei* described here demonstrates that this organism has the potential to exhibit novel behaviours in the CF host, including the development of chronic infection. Further analysis of *B. pseudomallei* isolates from those CF patients with persisting infection may inform on the key mechanisms contributing to bacterial persistence [7]. As a result of this analysis suggest the following recommendations:

1. Clinicians should have a high index of suspicion for *B. pseudomallei* infection for CF patients living in or returning from areas where it is endemic. Suspicion should be heightened when fever and or pneumonia occurs. Close liaison with the CF microbiology laboratory is important.
2. Eradication of *B. pseudomallei* infection should be attempted for CF patients when this pathogen is first isolated.
3. Initial intravenous therapy should include a minimum of two weeks intravenous ceftazidime (and or meropenem if severe sepsis). Consideration should be given to extending the duration of intravenous in those CF patients who are persistently culture-positive on therapy. We also recommend addition of oral/intravenous TMP/SMX from the onset of therapy and this should continue where possible for three months, with regular clinical monitoring for potential adverse effects including renal and hepatic dysfuntion, bone marrow toxicity and potentially life-threatening skin reactions including DRESS syndrome (drug reaction with eosinophilia and systemic symptoms).
4. Travel should be avoided to northern Australia or south-east Asia during the monsoonal season, with particular care to minimise exposure to wet season soils and rain in resident patients [20].
5. Person-to-person transmission of *B. pseudomallei* is generally thought not to occur. However one case of siblings who developed infection with identical strains raises the possibility of cross-infection in CF and should carefully consider the risks and benefits of segregation of patients who have isolated this organism.

## Additional files

Additional file 1:
**online search strategy.** (DOC 23 kb) Additional file 2:
**clinical data entry sheet.** (DOC 77 kb)

## Abbreviations

BMI: Body mass index
*B. pseudomallei*: *Burkholderia pseudomallei*
CF: Cystic fibrosis
CFTR: Cystic Fibrosis Transmembrane Conductance Regulator
FEV1: Forced expiratory volume in 1 second
TMP/SMX: Trimethoprim/sulfamethoxazole
