# Supplementary material for: An international, multicentre evaluation and description of Burkholderia pseudomallei infection in cystic fibrosis
Source: BMC Pulm Med. 2015 Oct 9;15:116. doi: 10.1186/s12890-015-0109-9 (PMC4600338; doi:10.1186/s12890-015-0109-9)
Supplement: Additional file 1: — online search strategy. (DOC 23 kb) [file 12890_2015_109_MOESM1_ESM.doc]

Database: Embase <1974 to 2014 August 26>

Search Strategy:

--------------------------------------------------------------------------------

1 *cystic fibrosis/ (34545)

2 *burkholderia pseudomallei/ (1157)

3 *melioidosis/ (1452)

4 2 or 3 (2163)

5 1 and 4 (8)

6 Burkholderia pseudomallei/ (2243)

7 1 and 6 (16)

8 7 not 5 (9)

9 from 8 keep 1-2,4-5,7 (5)

10 Melioidosis/ (1972)

11 1 and 10 (6)

***************************

**Online supplement – EMBASE search strategy**
