# Supplementary material for: An international, multicentre evaluation and description of Burkholderia pseudomallei infection in cystic fibrosis
Source: BMC Pulm Med. 2015 Oct 9;15:116. doi: 10.1186/s12890-015-0109-9 (PMC4600338; doi:10.1186/s12890-015-0109-9)
Supplement: Additional file 2: — clinical data entry sheet. (DOC 77 kb) [file 12890_2015_109_MOESM2_ESM.doc]

**CF BURKHOLDERIA PSEUDOMALLEI – DATA COLLECTION FORM**

| **CF CENTRE** | **CITY/COUNTRY** | **PATIENT NUMBER** | **DOB** |
| --- | --- | --- | --- |
|  |  |  |  |

**PRIMARY PHYSICIAN: __________________________________________**

**EMAIL:­­­­­­­­­­­­­­­­­_________________________________________________________**

**PHONE (INCL COUNTRY CODE)__________________________________**

| **PATIENT DATA** |
| --- |

1. **BASELINE CLINCAL DATA – AT TIME OF FIRST ISOLATE**

| **Date of first isolate** |  |
| --- | --- |
| Gender | male female |
| Ethnicity  (e.g Caucasian) |  |
| Height (metres) |  |
| Weight (kg) |  |
| Alcohol Intake | No  Yes Estimated daily ETOH intake (grams) ___________ |
| Current smoker | No  Yes Estimated pack year history _______________ |
| Rheumatic Heart Disease | Yes  No |
| Congestive cardiac failure | Yes  No |
| CFTR Mutation | allele 1 _________  allele 2 ­_________ not known |

1. **LUNG DISEASE – AT TIME OF FIRST ISOLATE**

| **FEV1** | **absolute (L) __________**  **percent predicted ___________** |
| --- | --- |
| Bacterial and fungal pathogens | Pseudomonas (non mucoid)  Pseudomonas (mucoid)  Staph aureus (methicillin sensitive)  Staph aureus (methicillin resistant)  Burkolderia cepacia  Aspergillus  Other (please specify e.g Stenotrophomonas, Achromobacter, non-tuberculous mycobacteria, fungi etc.)  ­­­­­­­­­­­­­­­­­­­­­­­­­_____________________________________________________ |
| Allergic Bronchopulmonary Aspergillosis (ABPA) | Yes – ever previously formally diagnosed  No – never previously formally diagnosed |

**3. NON-PULMOMARY CF MANIFESTATIONS – AT FIRST ISOLATE**

| **Pancreatic exocrine insufficiency** | **yes**  **no** |
| --- | --- |
| Pancreatic endocrine function | CF related diabetes --- Insulin treated  Not insulin treated  ---  HbA1C (if known) _____________  Impaired fasting glucose/glucose tolerance  Normal glucose tolerance  Not known |
| CF liver disease | Cirrhosis on imaging (MRI, CT, Ultrasound)  Portal Hypertension (oesophageal varicies, portal-systemic collateral vessels, splenogmegaly)  Abnormal liver function tests without structural liver diease |

**4. DIAGNOSTIC FEATURES**

| **Clinical Features**  **at time of presumed acquisition/first isolate** | **fevers**  **cough**  **increased sputum**  **weight loss**  **night sweats**  **chest pain**  **impaired glycaemic control**  **incidental finding on sputum surveillance**  **other (please specify)**  **___________________________________________________** |
| --- | --- |
| Radiological features at time of first isolate | New changes – please specifiy (e.g consolidation, deteriorating bronchiectasis, reticulonodular changes etc.)    Stable imaging  None performed |
| Acquisition | Location of presumed exposure ________________________  (e.g Northern Australia)  Approximate date of presumed exposure ________________  Time spent in area of exposure:  weeks  months  years |

1. **TREATMENT RECEIVED**

| **Treatment** | **Was eradication attempted:**  **Yes**  **No If YES……**  **Ambulatory treatment**  **Inpatient treatment**  **Both**  **If inpatient treatement - Was the patient treated in respiratory isolation?**  **If ambulatory treatment - Was the patient segregated from other patients?**  **Parenteral antibiotics?**  **Duration _________________________________________________**    **Agent(s) _________________________________________________**  **_________________________________________________**    **Oral antibiotics?**  **Duration _________________________________________________**  **Agent(s) ___________________________________**  **___________________________________**  **Ongoing suppressive therapy? (Please specify e.g sulfamethoxazole/trimethoprim)**  **_____________________________________________**  **_____________________________________________** |
| --- | --- |

1. **CLINICAL TRAGECTORY POST ACQUISITION**

| **Date last clinical review** | **--/--/----** |
| --- | --- |
| Most recent FEV1  Date: __/__/____ | absolute (ml) _______________  percent predicted____________ |
| Most recent weight (kg)  Date: __/__/____ | Weight |
| Most recent Height (m)  Date __/__/____ | Height |
| Ongoing infection  (i.e ongoing sputum isolates) | Yes ----  Number positive isolates _________  Unknown  No |
| If no ongoing infection | Date earliest consistently negative sputum  Date of last consistently negative sputum  Number of negative sputa __________________ |
| Mortality | Date of death ___________________________ |
| Pulmonary Transplantation | Date of transplantation ___________________  Persistent infection post transplantation  Ongoing suppressive antiobiotic treatment post transplantation  Yes (please specify) __________________  No  No – previously cleared |
| Clinical impression | Accelerated decline post acquisition  Clinically stable post accquisition |
